# Last Word on Viewpoint: Cardiac “potential energy” estimation: ambiguous and subjective

**DOI:** 10.1152/japplphysiol.00454.2024

**Published:** 2024-07-01

**Authors:** June-Chiew Han, Toan Pham, Andrew J. Taberner, Kenneth Tran

**Affiliations:** ^1^Auckland Bioengineering Institute, The University of Auckland, Auckland, New Zealand; ^2^Department of Engineering Science and Biomedical Engineering, The University of Auckland, Auckland, New Zealand

**Keywords:** efficiency, end-systolic, force-length relation, pressure volume area, work-loop

Cardiac “potential energy” (PE, which we label U) has been estimated from the pressure-volume (P-V) [and force-length (F-L)] plane, as the “triangle” area at the lower-left corner (Fig. 1 in our Viewpoint in Ref. 1). Given that cardiac end-systolic (ES) P-V (and F-L) relation is contraction mode-dependent, the upper boundary of the “triangle” area is often subjective. For ejecting contractions, the upper boundary depends on the loading conditions of the work-loop, which violates a key assumption of Suga’s “pressure-volume-area” (PVA) concept. This is the basis for the ambiguity in the estimation of “potential energy” under work-loop contractions, which has consequences on the assessment and understanding of cardiac efficiency.

We are encouraged by the comments of Durço et al. (2) that support our analysis of the uncertainty in estimating U, which presents practical implications for both basic and clinical research.

We agree with Sogard and Mickeborough (3) on the need to consider various factors in “understanding an individual’s work-loops” to accurately provide “the estimation of stroke work” (W). When W is approximated by a “rectangle” on the P-V (and F-L) axis, as is quantified from noninvasive measurements as the product of mean arterial pressure and stroke volume, it can distort the true estimation of W. In contrast, invasive measurements of both P and V, and direct measurements of F and L in isolated muscles as in our experiments (4–7), yield a true representation of work-loop, where the area of the loop is accurately quantified as W by integrating P over V (and F over L). However, in our Viewpoint (1), we focus on U and show that for any work-loop, PE (or U) cannot be obtained without ambiguity. Thus, U has been estimated in the field using various methods, resulting in its various estimates. These various methods, which we categorise into three methods, differ from one another in how they define the upper boundary of the “triangle” area and are not entirely consistent with the Suga's PVA concept.

Suga specified that:

1)  The end-systolic point of a work-loop meets the end-systolic point of an isometric contraction at the same end-systolic length.
2)  The upper boundary of PE is the isometric F-L relation.
3)  In a series of work-loops, the upper boundary of PE is unique and it is the isometric F-L relation.
4)  The upper boundary of PE intersects the dead-space volume, V_o_ (or dead-space length, L_ds_).

All three methods violate at least one of these criteria.

Many cardiac investigators over half a century have incorrectly believed that the upper boundary of the “triangle” area (however, PE is estimated) is the isometric F-L relation. Our critique of Suga’s potential energy concept in the Viewpoint (1) stems from theoretical (6, 8) and experimental (4–7) evidence that reinforces the contraction mode-dependence of the cardiac P-V (and F-L) relation that was revealed by Otto Frank over a century ago (9).

With great respect to the comments of Marocolo and Souza (10), we submit that we are not proposing a new approach but rather providing a summary of the various methods that have been used to attempt to quantify U. U should not have been in the numerator of cardiac efficiency in the first place. For over a century since the 1910s, the numerator of cardiac efficiency has consisted only of the actual, measurable, mechanical work (W) performed by contracting muscle.

We hope that our Viewpoint (1) will discourage the field from using the “potential energy” concept while simultaneously encouraging the field to, at least, think more seriously about the ambiguity and adverse implications associated with using the concept to understand the fundamentals of cardiac energetics and efficiency.

## GRANTS

This study was supported by the Health Research Council of New Zealand through Sir Charles Hercus Health Research Fellowship Grants (20/011 and 21/116, awarded to J.-C.H. and K.T., respectively), Explorer Grant (21/758, awarded to J.-C.H.) and Emerging Researcher First Grant (21/653, awarded to T.P.), the Royal Society of New Zealand through Marsden Project Grant (MFP-UOA2206, awarded to J.-C.H.) and James Cook Research Fellowship (awarded to A.J.T.), and the Heart Foundation of New Zealand through Project Grant (1929, awarded to J.-C.H.) and Research Fellowship Grant (1896, awarded to T.P.).

## DISCLOSURES

No conflicts of interest, financial or otherwise, are declared by the authors.

## AUTHOR CONTRIBUTIONS

J.-C.H. conceived and designed research; J.-C.H. performed experiments; J.-C.H. analyzed data; J.-C.H., T.P., A.J.T., and K.T. interpreted results of experiments; J.-C.H. prepared figures; J.-C.H. drafted manuscript; J.-C.H., T.P., A.J.T., and K.T. edited and revised manuscript; J.-C.H., T.P., A.J.T., and K.T. approved final version of manuscript.
